# Correction: Approach-Induced Biases in Human Information Sampling

**DOI:** 10.1371/journal.pbio.1002618

**Published:** 2017-11-30

**Authors:** Laurence T. Hunt, Robb B. Rutledge, W. M. Nishantha Malalasekera, Steven W. Kennerley, Raymond J. Dolan

In Fig 2, the title for panel C is incorrect. The panel title should read “Positive evidence approach: MULTIPLY BIG minus MULTIPLY SMALL” rather than “Sampling the favorite: MULTIPLY BIG minus MULTIPLY SMALL”. Please see the corrected Fig 2 here.

**Fig 2:**
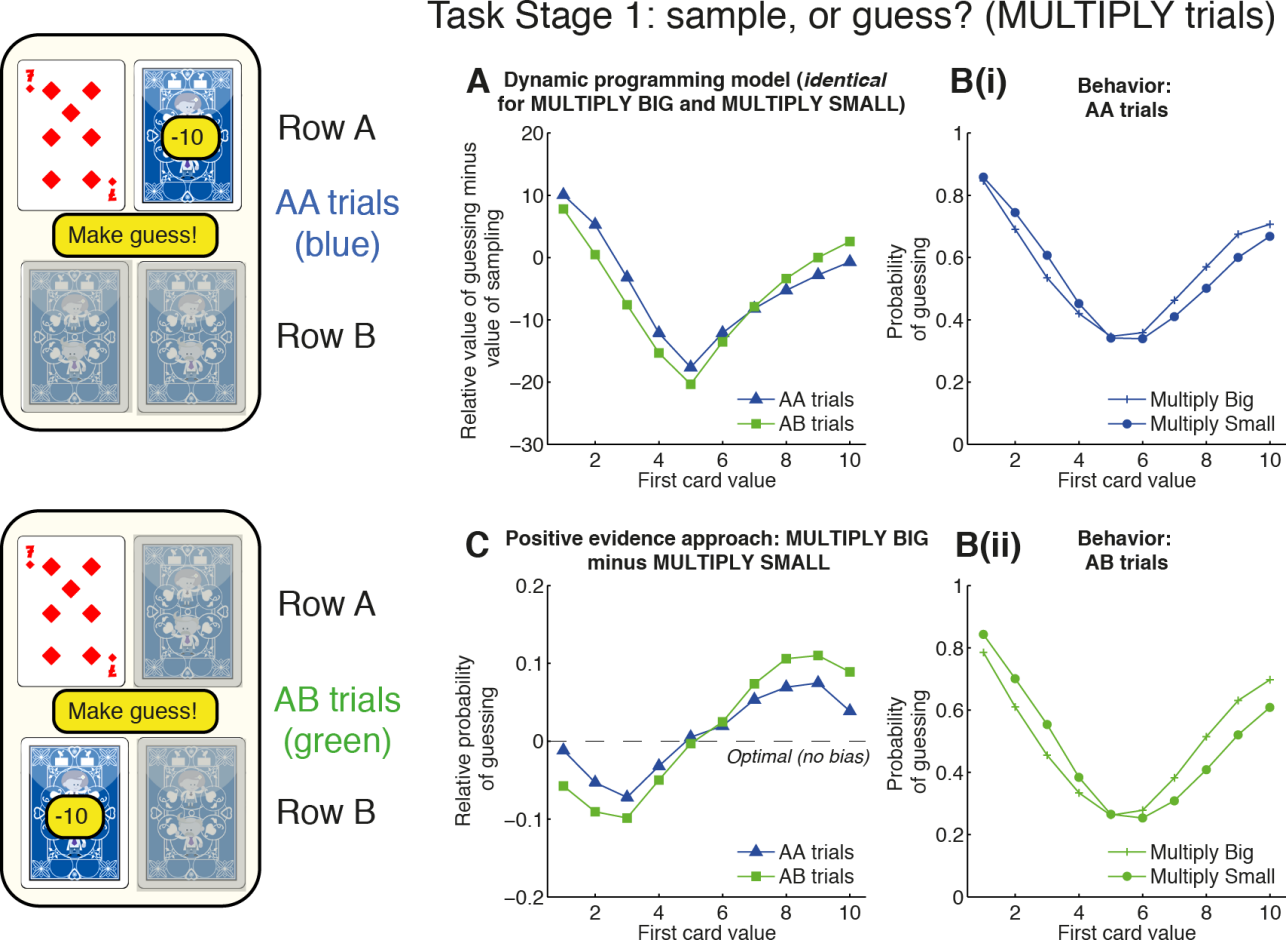
Positive evidence approach bias at Task Stage 1. At Task Stage 1, subjects decide whether to make a guess or pay 10 points to sample. The available card to sample may be on the same row (“AA trials”) or the opposite row (“AB trials”) as the first card. **(A)** Model predictions. The relative expected value (in points) of guessing versus sampling from the dynamic programming model in the MULTIPLY conditions. Mid-valued cards make it more valuable to sample, whereas extreme-valued cards make it more valuable to guess. There is a weaker influence of the location of available information (compare “AA trials” versus “AB trials”). Crucially, optimal behavior is identical for both MULTIPLY BIG and MULTIPLY SMALL conditions. **(B)** Subject behavior. The probability of guessing in both conditions shows a broad similarity to the predictions of the dynamic programming model, but behavior in MULTIPLY BIG and MULTIPLY SMALL shows systematic differences. (See S1 Fig for AA and AB trials plotted together, rather than MULTIPLY BIG and MULTIPLY SMALL.) **(C)** Positive evidence approach bias is revealed by subtracting the MUTLIPLY SMALL condition from the MULTIPLY BIG condition. Subjects are more likely to guess early if they have seen evidence that supports them approaching row A rather than avoiding it. This effect is strengthened in AB trials, in which subjects only have the opportunity to sample further information about row B. See also S2 Fig and S3 Fig for other conditions. Data for reproducing all analyses is freely available for download from Dryad.
