# Supplementary figures and images for: Approach-Induced Biases in Human Information Sampling
Source: PLoS Biol. 2016 Nov 10;14(11):e2000638. doi: 10.1371/journal.pbio.2000638 (PMC5104460; doi:10.1371/journal.pbio.2000638)

**Behavior:**  
**MULTIPLY BIG**

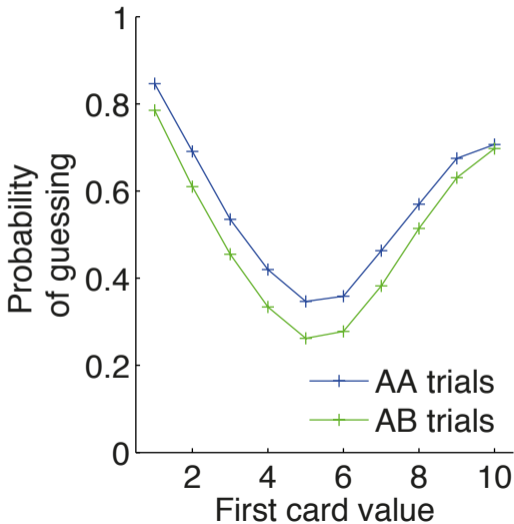

**Behavior:**  
**MULTIPLY SMALL**

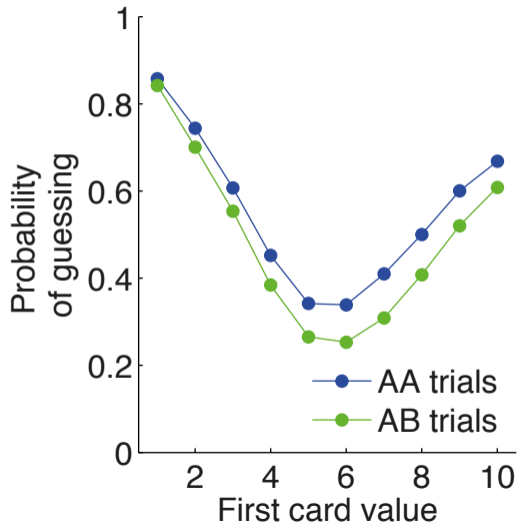

Supplement: S1 Fig — Data are the same as in main Fig 2B, but are replotted with AA and AB trials on top of each other to facilitate comparison with dynamic programming model predictions. (PDF) [file pbio.2000638.s001.pdf]

# Task Stage 1: sample, or guess? (SINGLE CARD trials)

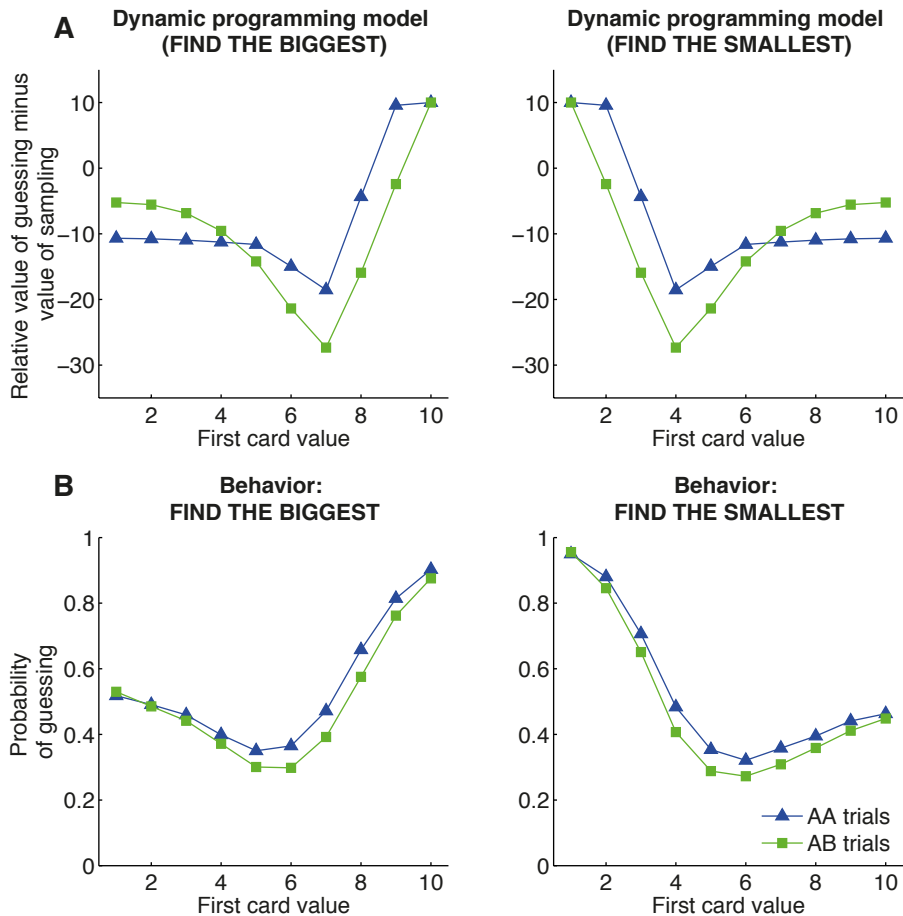

Supplement: S3 Fig — Note that in single card trials, the same card value carries different amounts of information between the two conditions (hence part A is split into two plots). As such, there is no direct equivalent for the positive evidence approach bias. (PDF) [file pbio.2000638.s003.pdf]

# Making guess, after task stage 1: which row chosen? (SINGLE CARD trials)

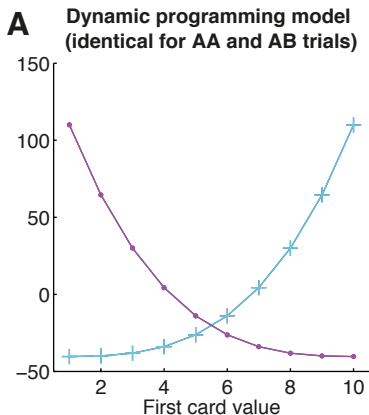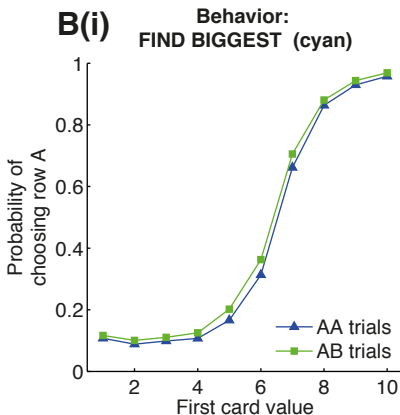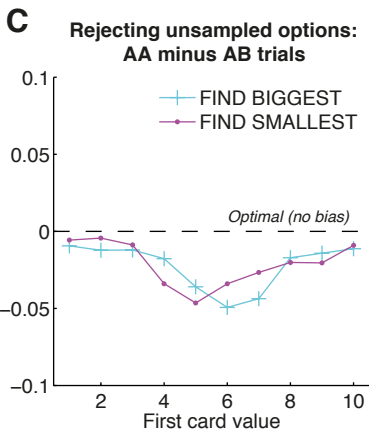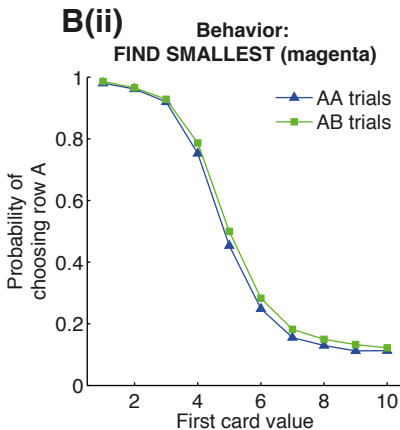

Supplement: S5 Fig — (PDF) [file pbio.2000638.s005.pdf]

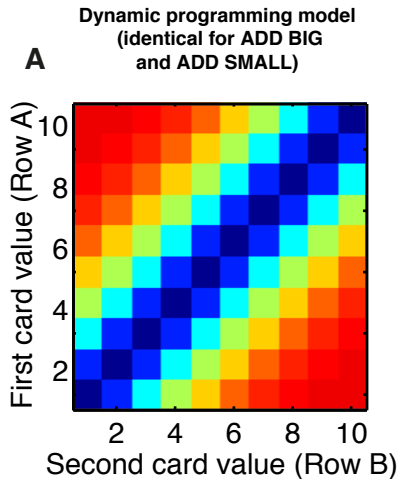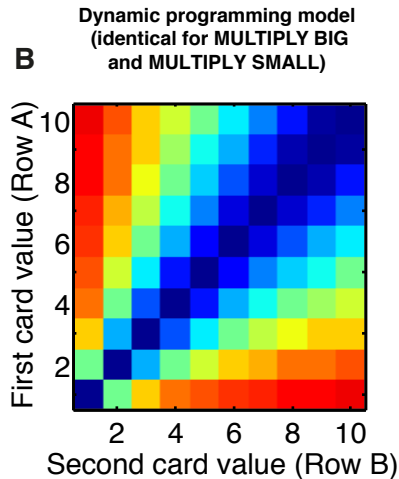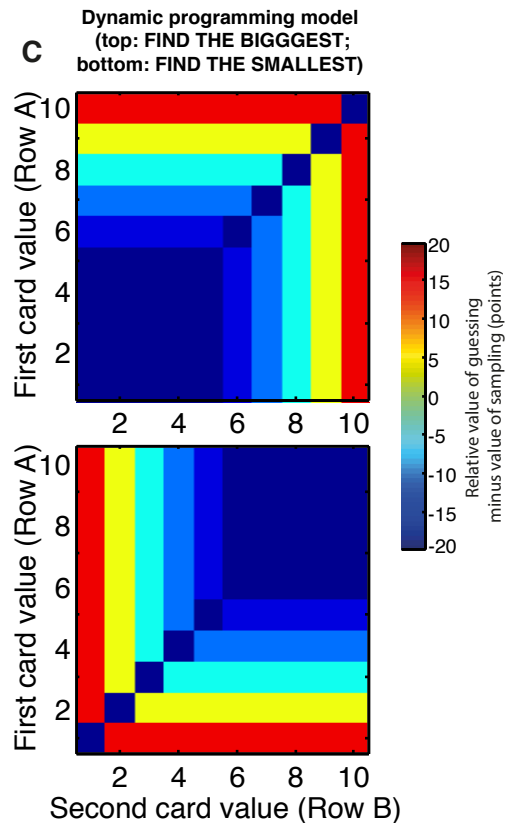

Supplement: S8 Fig — (A) ADD conditions, where predictions are identical for ADD BIG and ADD SMALL. (B) MULTIPLY conditions, where predictions are identical for MULTIPLY BIG and MULTIPLY SMALL. (C) SINGLE CARD conditions. Top row = FIND THE BIGGEST condition, bottom row = FIND THE SMALLEST condition. (PDF) [file pbio.2000638.s008.pdf]

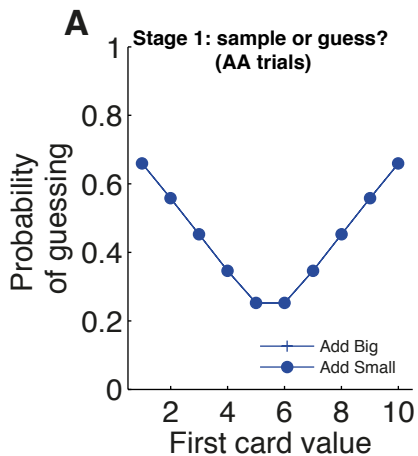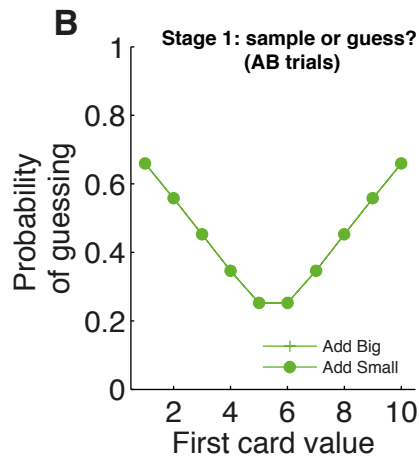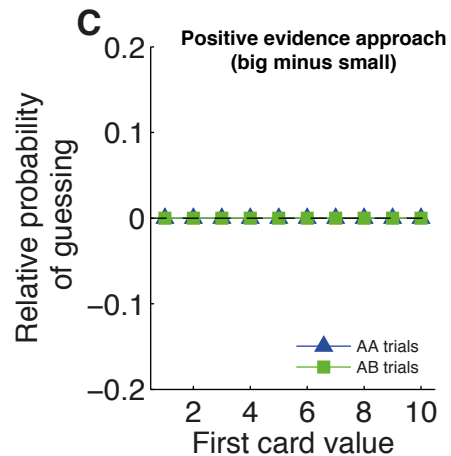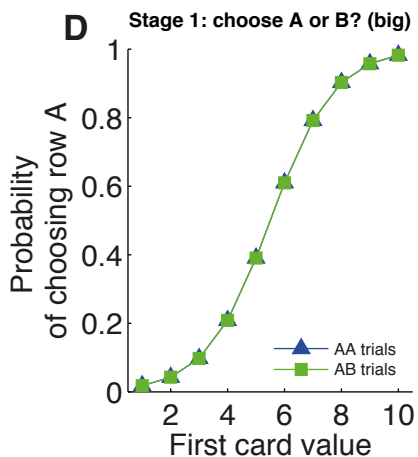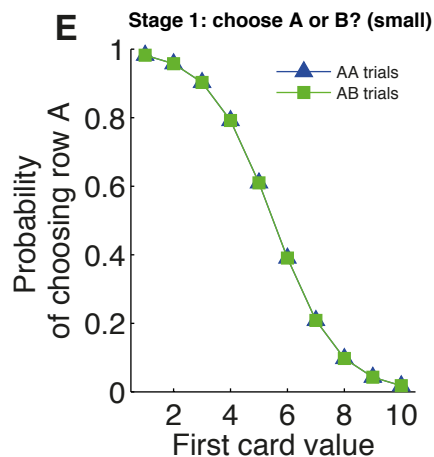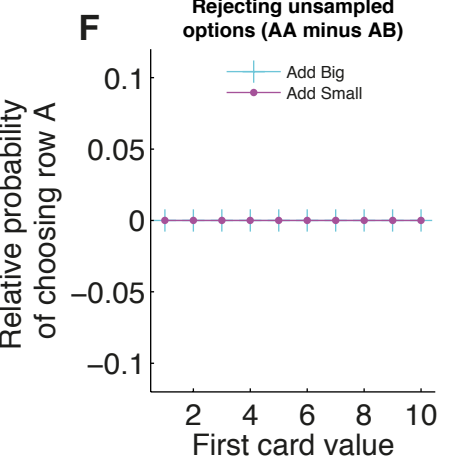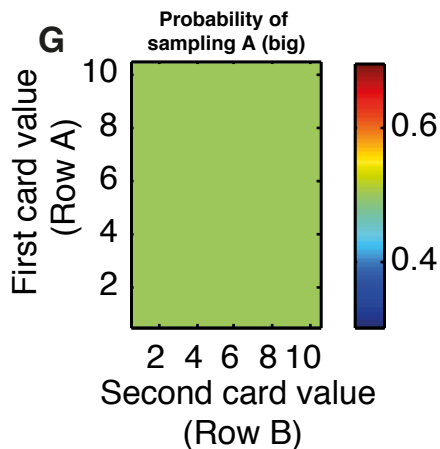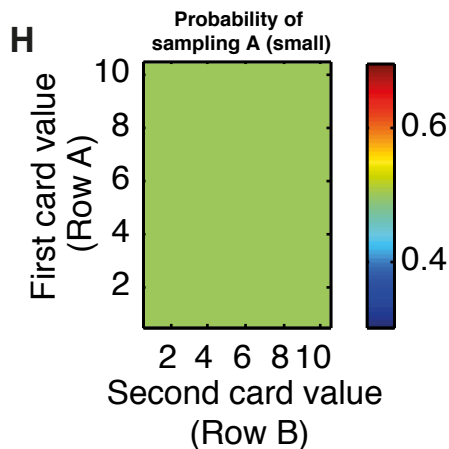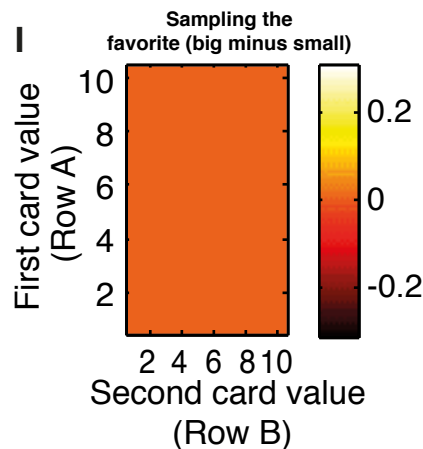

Supplement: S9 Fig — Data is plotted as in main Fig 5. (A) Predicted probability of guessing at stage 1 for AA trials and (B) AB trials in ‘add’ condition. (C) Predicted ‘positive evidence approach’ bias. (D) Predicted probability of choosing row A, having chosen to guess, at stage 1, for ‘add big’ and (E) ‘add small’ conditions. (F) Predicted ‘rejecting unsampled options’ bias. (G) Predicted probability of sampling row A in ‘big’ and in (H) ‘small’ conditions. (I) Predicted ‘sampling the favorite’ bias. (PDF) [file pbio.2000638.s009.pdf]

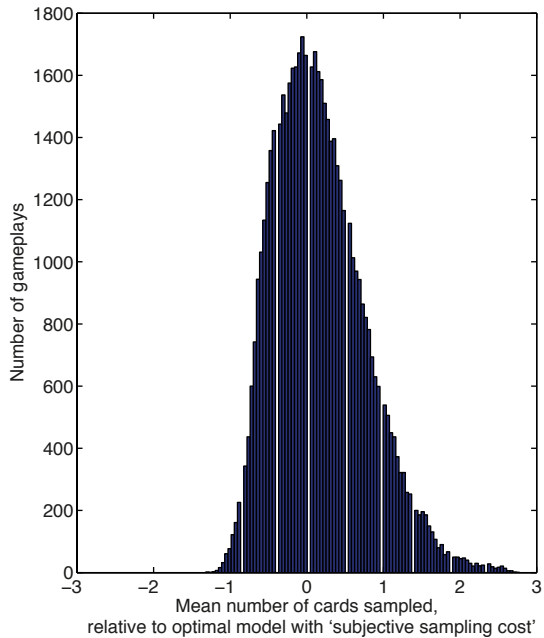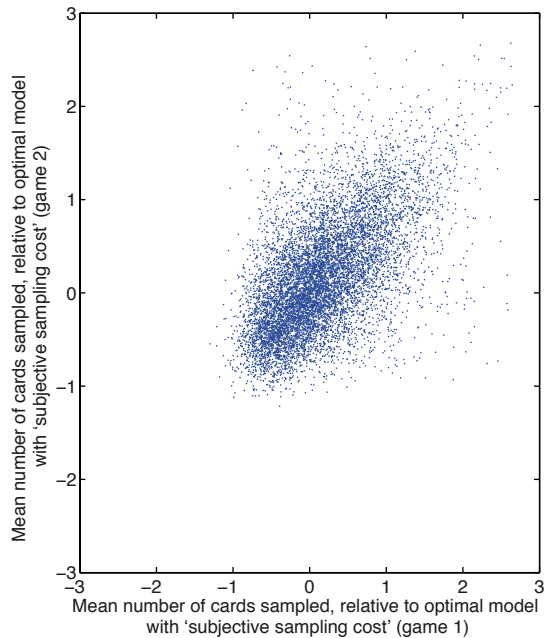

Supplement: S10 Fig — The mean of the distribution of the number of cards sampled relative to the model (left panel) now lies close to 0. (PDF) [file pbio.2000638.s010.pdf]

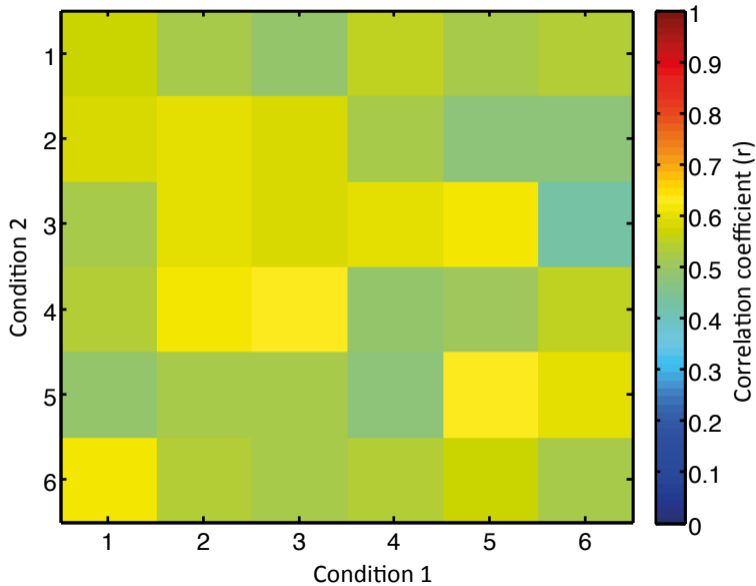

Supplement: S11 Fig — Along the bottom of the matrix is the condition experienced in the first 11 trials of gameplay 1 (1 = FIND BIGGEST, 2 = FIND SMALLEST, 3 = ADD BIG, 4 = ADD SMALL, 5 = MULTIPLY BIG, 6 = MULTIPLY SMALL), whilst along the left of the matrix is the condition experienced in the first 11 trials of gameplay 2. The color of the heatmap reflects the correlation coefficient between information sampling (relative to the optimal model) across the two gameplays (as in main Fig 7B). (PDF) [file pbio.2000638.s011.pdf]
