# Supplementary material for: Approach-Induced Biases in Human Information Sampling
Source: PLoS Biol. 2016 Nov 10;14(11):e2000638. doi: 10.1371/journal.pbio.2000638 (PMC5104460; doi:10.1371/journal.pbio.2000638)
Supplement: S2 Fig — Note that there is now no difference between normative model predictions for AA vs. AB trials. (PDF) [file pbio.2000638.s002.pdf]

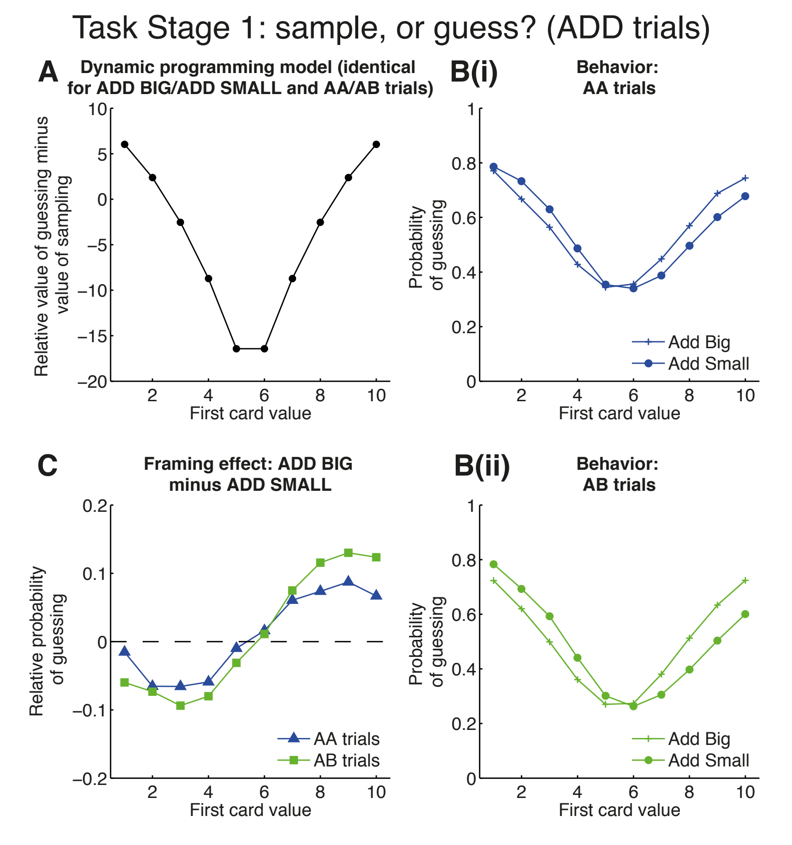

Task Stage 1: sample, or guess? (ADD trials)
A
Dynamic programming model (identical for ADD BIG/ADD SMALL and AA/AB trials)
Relative value of guessing minus value of sampling
First card value
B(i)
Behavior: AA trials
Probability of guessing
Add Big
Add Small
First card value
C
Framing effect: ADD BIG minus ADD SMALL
Relative probability of guessing
AA trials
AB trials
First card value
B(ii)
Behavior: AB trials
Probability of guessing
Add Big
Add Small
First card value
