# Supplementary material for: Approach-Induced Biases in Human Information Sampling
Source: PLoS Biol. 2016 Nov 10;14(11):e2000638. doi: 10.1371/journal.pbio.2000638 (PMC5104460; doi:10.1371/journal.pbio.2000638)
Supplement: S4 Fig — (PDF) [file pbio.2000638.s004.pdf]

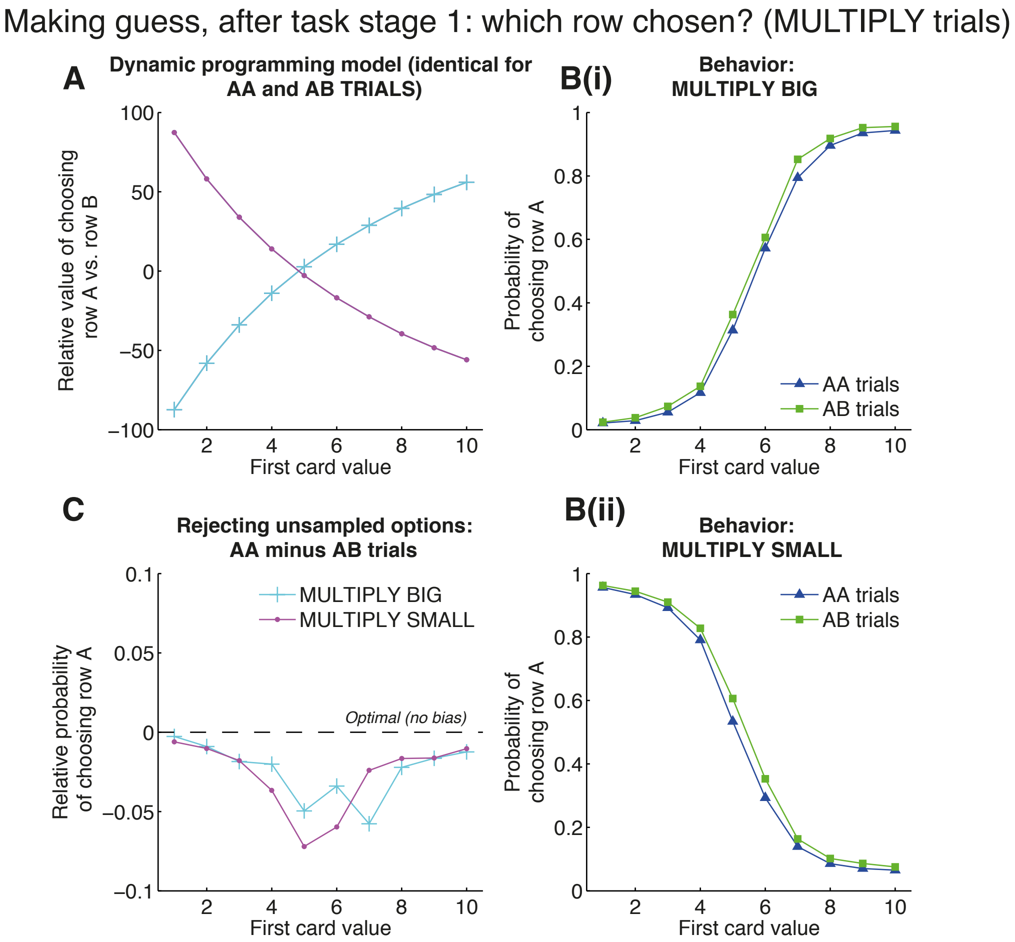

Making guess, after task stage 1: which row chosen? (MULTIPLY trials)
A
Dynamic programming model (identical for AA and AB TRIALS)
Relative value of choosing row A vs. row B
100
50
0
−50
−100
2
4
6
8
10
First card value
B(i)
Behavior: MULTIPLY BIG
Probability of choosing row A
1
0.8
0.6
0.4
0.2
0
AA trials
AB trials
First card value
C
Rejecting unsampled options: AA minus AB trials
MULTIPLY BIG
MULTIPLY SMALL
Optimal (no bias)
Relative probability of choosing row A
0.1
0.05
0
−0.05
−0.1
First card value
B(ii)
Behavior: MULTIPLY SMALL
Probability of choosing row A
AA trials
AB trials
First card value
