# Supplementary material for: Approach-Induced Biases in Human Information Sampling
Source: PLoS Biol. 2016 Nov 10;14(11):e2000638. doi: 10.1371/journal.pbio.2000638 (PMC5104460; doi:10.1371/journal.pbio.2000638)
Supplement: S6 Fig — Note that there is no relative advantage for sampling row A over row B in the normative model (part A). (PDF) [file pbio.2000638.s006.pdf]

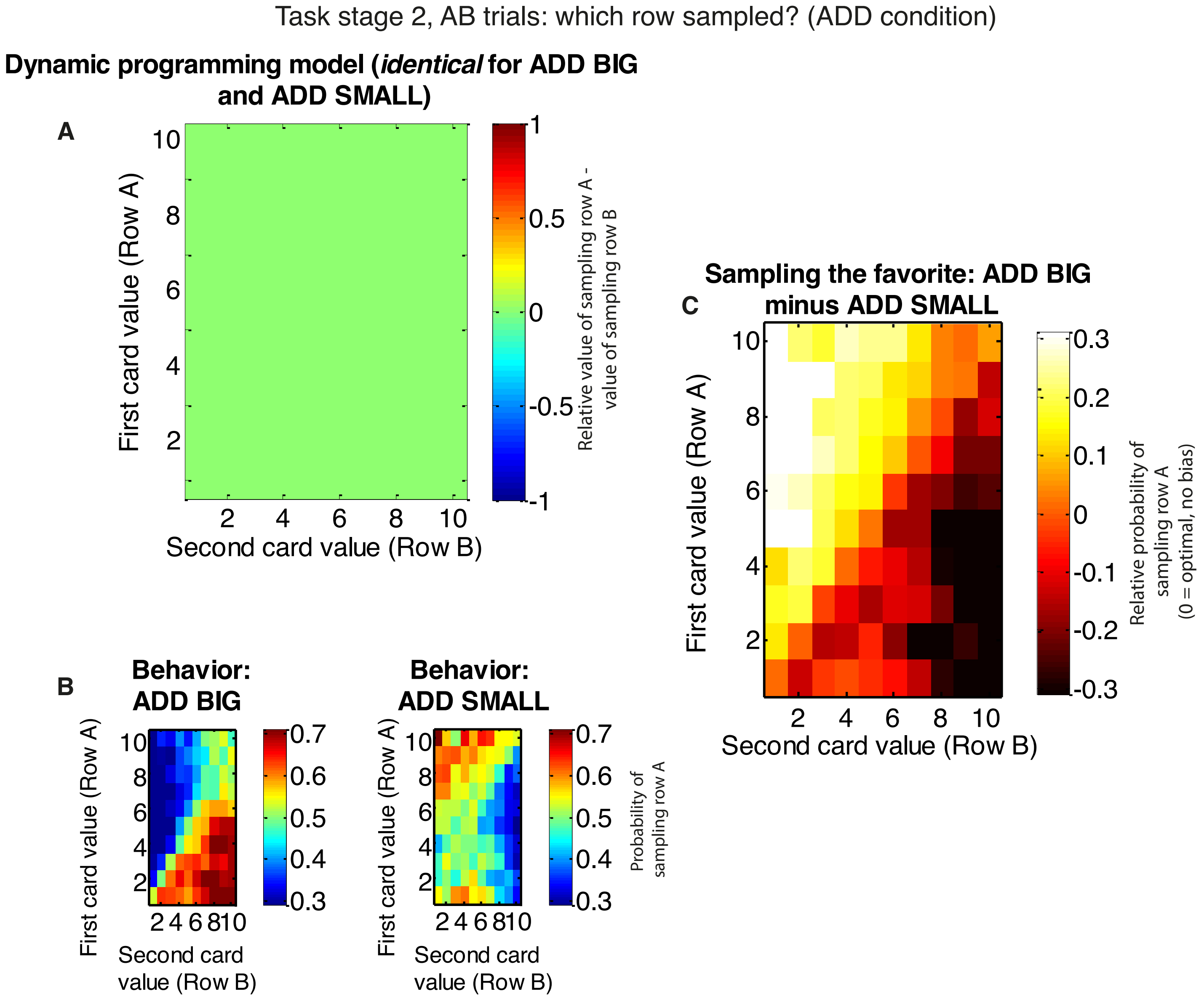

Task stage 2, AB trials: which row sampled? (ADD condition)
Dynamic programming model (identical for ADD BIG and ADD SMALL)
A
First card value (Row A)
Second card value (Row B)
Relative value of sampling row A - value of sampling row B
B
Behavior: ADD BIG
Behavior: ADD SMALL
First card value (Row A)
Second card value (Row B)
Probability of sampling row A
C
Sampling the favorite: ADD BIG minus ADD SMALL
First card value (Row A)
Second card value (Row B)
Relative probability of sampling row A (0 = optimal, no bias)
