# Supplementary material for: Approach-Induced Biases in Human Information Sampling
Source: PLoS Biol. 2016 Nov 10;14(11):e2000638. doi: 10.1371/journal.pbio.2000638 (PMC5104460; doi:10.1371/journal.pbio.2000638)
Supplement: S7 Fig — Note that in single card trials, the same card value carries different amounts of information between the two conditions (hence part A is split into two plots). As such, there is no direct equivalent for the sampling the favourite bias. (PDF) [file pbio.2000638.s007.pdf]

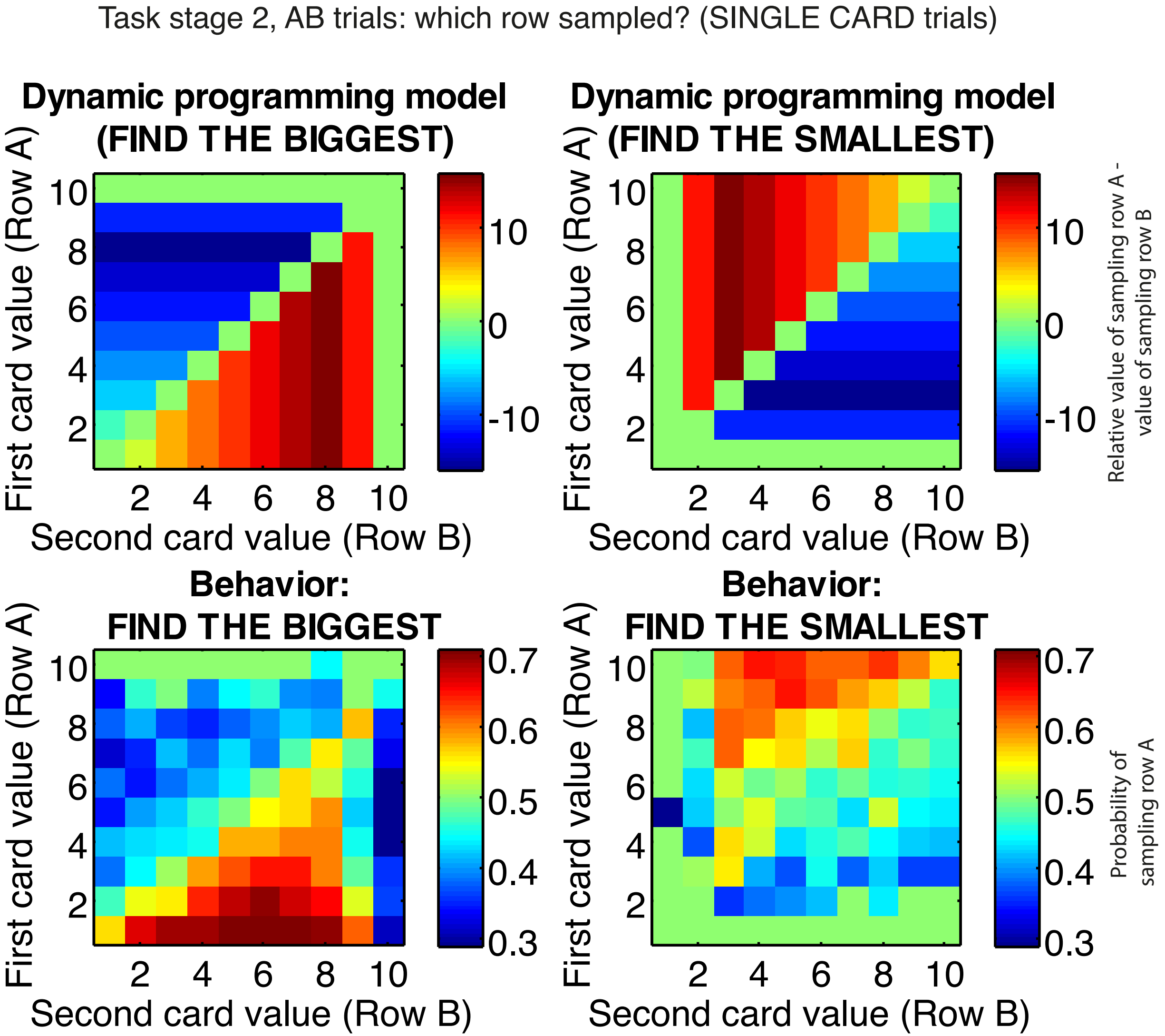
Task stage 2, AB trials: which row sampled? (SINGLE CARD trials)
Dynamic programming model (FIND THE BIGGEST)
Dynamic programming model (FIND THE SMALLEST)
Behavior: FIND THE BIGGEST
Behavior: FIND THE SMALLEST
First card value (Row A)
Second card value (Row B)
Relative value of sampling row A - value of sampling row B
Probability of sampling row A
10
0
-10
0.7
0.6
0.5
0.4
0.3
2
4
6
8
10
