# Supplementary material for: Approach-Induced Biases in Human Information Sampling
Source: PLoS Biol. 2016 Nov 10;14(11):e2000638. doi: 10.1371/journal.pbio.2000638 (PMC5104460; doi:10.1371/journal.pbio.2000638)
Supplement: S1 Table — (DOCX) [file pbio.2000638.s012.docx]

**Supplemental table S1: Maximum likelihood estimates of parametric model of subject behavior**

|  | ADD (Stage 1) | MULTIPLY (Stage 1) | ADD (Stage 2) | MULTIPLY (Stage 2) |
| --- | --- | --- | --- | --- |
| τ | 1.6660 | 1.1221 | 2.8949 | 4.0448 |
| β_1_ | 0.7417 | 0.5126 | 0.8534 | 1.2028 |
| β_2_ | 3.1108 | 2.0920 | 4.9238 | 6.8225 |
| β_3_ | 3.3886 | 2.4596 | 15.4195 | 22.3263 |
| β_4_ | 0.0669 | 0.0714 | N/A | N/A |
| β_5_ | 0.1035 | 0.0450 | N/A | N/A |
| β_6_ | N/A | N/A | 0.1856 | 0.2858 |
