# Supplementary material for: Approach-Induced Biases in Human Information Sampling
Source: PLoS Biol. 2016 Nov 10;14(11):e2000638. doi: 10.1371/journal.pbio.2000638 (PMC5104460; doi:10.1371/journal.pbio.2000638)
Supplement: S2 Table — (DOCX) [file pbio.2000638.s013.docx]

**Supplemental table S2: Sum of square errors for model fitting on left-out data during 10-fold nested cross-validation (mean ± SEM)**

|  | ADD (Stage 1) | MULTIPLY (Stage 1) | ADD (Stage 2) | MULTIPLY (Stage 2) |
| --- | --- | --- | --- | --- |
| Full model | 0.215 **±** 0.013 | 0.260 **±** 0.019 | 3.979 **±** 0.130 | 4.564 **±** 0.090 |
| Reduced model | 0.361 **±** 0.016 | 0.406 **±** 0.020 | 4.671 **±** 0.162 | 5.272 **±** 0.095 |
