# Supplementary material for: Approach-Induced Biases in Human Information Sampling
Source: PLoS Biol. 2016 Nov 10;14(11):e2000638. doi: 10.1371/journal.pbio.2000638 (PMC5104460; doi:10.1371/journal.pbio.2000638)
Supplement: S1 Text — (DOCX) [file pbio.2000638.s014.docx]

**Supplemental text: Instructions provided to subjects**

**Introduction:**

We often make choices involving risk. Sometimes bigger gains are made by taking a riskier approach, but sometimes playing it safe yields more long-term rewards. People differ substantially in their risk-taking behaviour.

In this card-turning game, you’ll decide whether it’s worth taking a risk in order to gain more points.

*[subject can read this information by touching ‘More info’, optional]:*

**More information:**

Many neuroscientists are interested in why we make certain decisions. Recently, it has become popular to borrow ideas from economics to explain both human behaviour and brain activity whilst choices are made.

Economists have built models of how humans should use information about risk to guide their decisions. They have also studied where human behaviour systematically deviates from these predictions.

Damage to certain brain regions, such as prefrontal cortex, impairs humans’ ability to make risky decisions accurately. Using brain imaging, scientists are beginning to uncover what role these regions might play in the intact brain.

*[subject touches ‘Play’, then sees this:]*

**How to Play:**

You will be playing a simple card game. For each hand, choose the winning row to gain points. You’ll be told before each level how to find the winning row.

To help you decide, touch a highlighted card to turn it over. This will cost you the number of points shown on the card back:


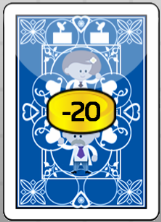


When you think you know which is the winning row, touch the ‘make a guess’ button and then tap on the row. Try to make as many points as possible!

*[subject touches ‘Start’, then sees a screen like this (differs depending upon condition):]*

**
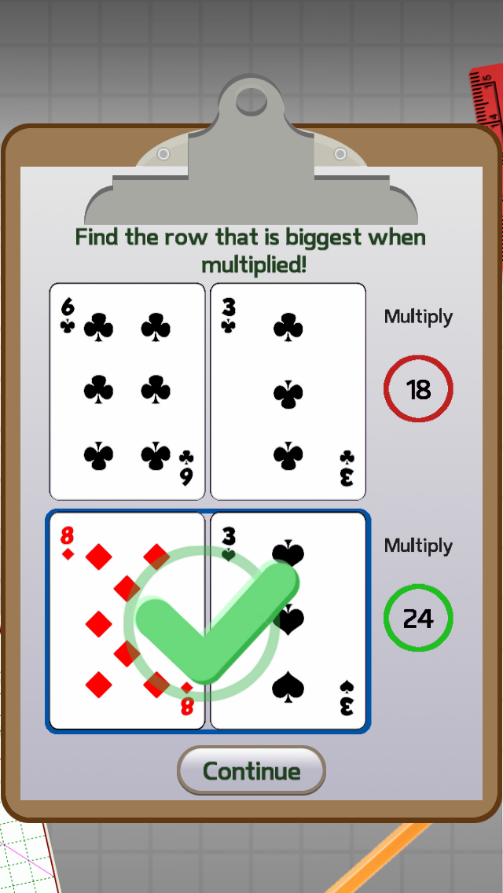
**

*[subject touches ‘Continue’. It flashes ‘Picture cards have been removed from the deck!’]*


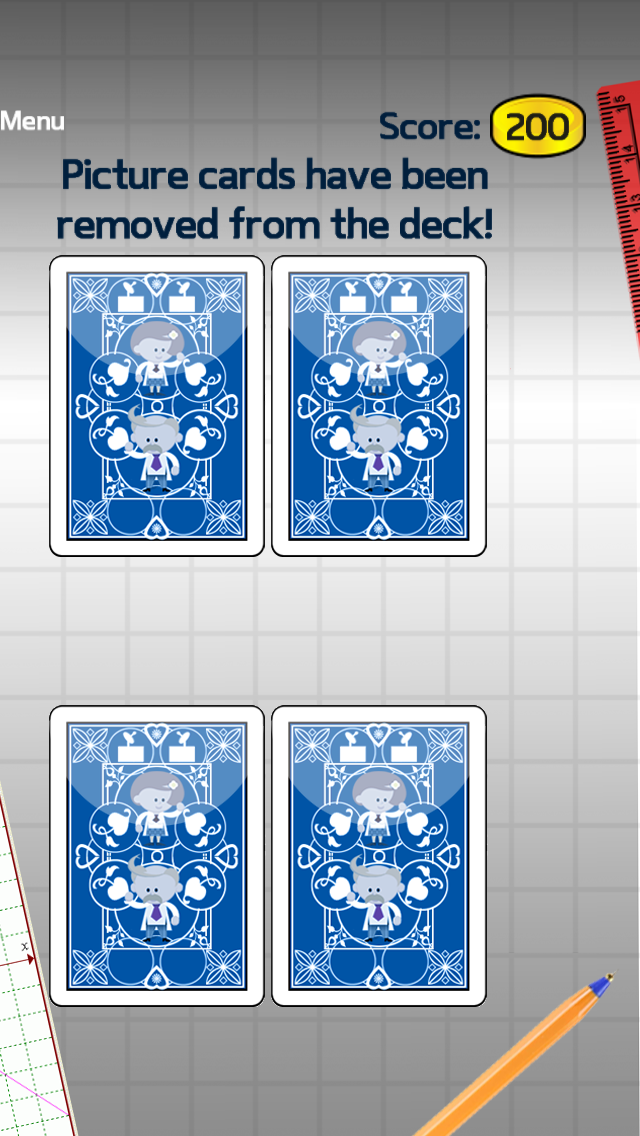


*[It then continues:]*

*
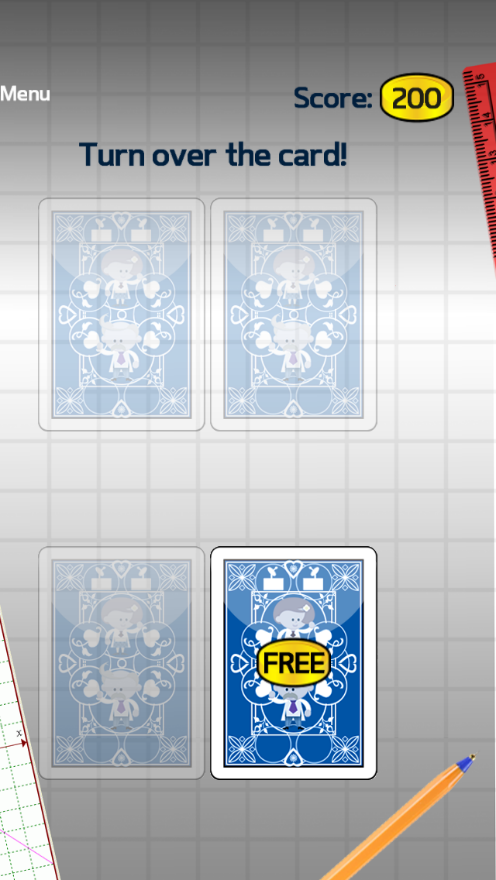
*

*[Subject touches ‘FREE’ to enter Task Stage 1:]*

*
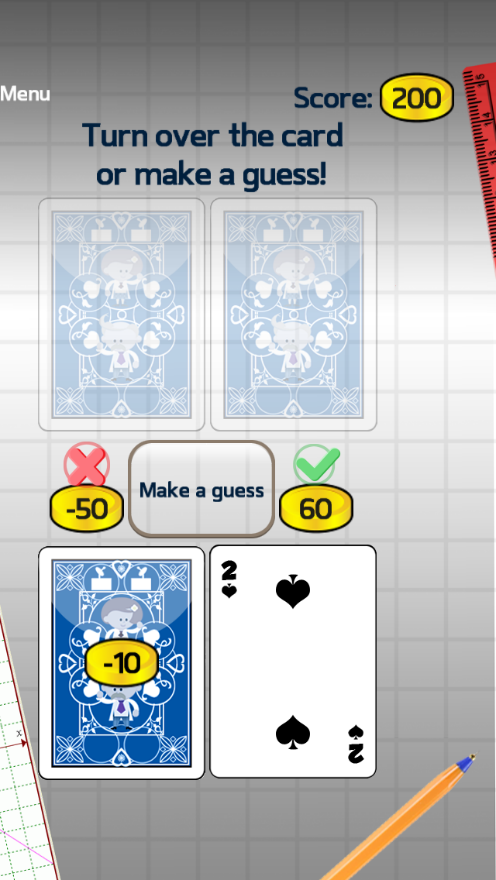
*

*[Subject touches ‘Make a Guess’ to enter Choice stage:]*

*
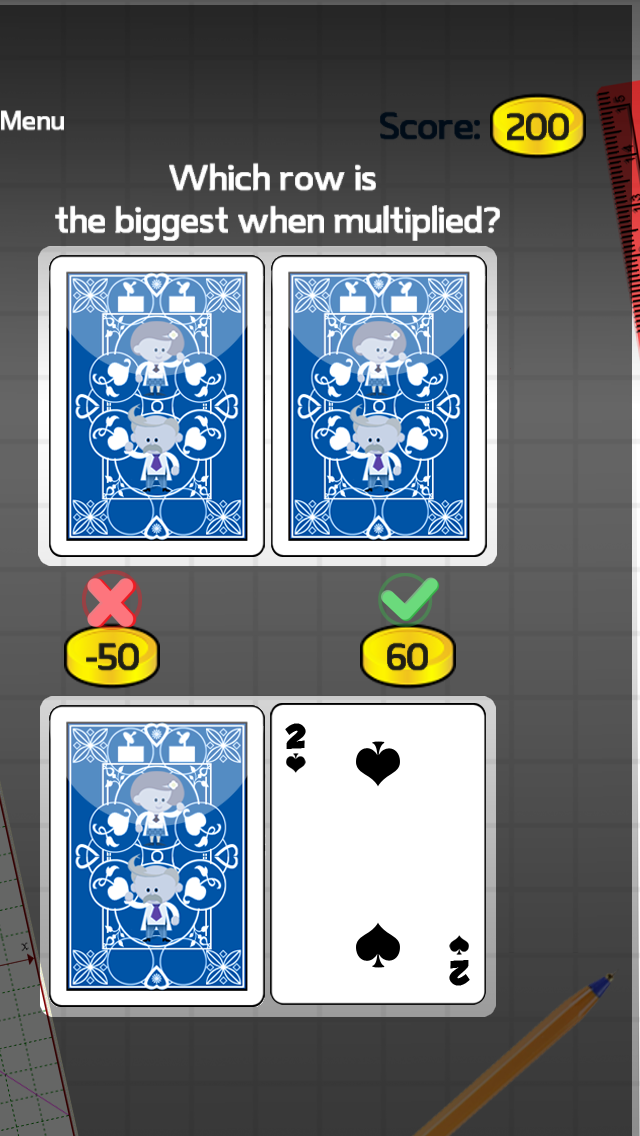
*

*[Subject touches top or bottom row to complete trial.]*
